# Case of Amaurosis of Both Eyes, of Twelve Months Standing

**Published:** 1816-06

**Authors:** H. John Rheineck, Von Embden

**Affiliations:** Surgeon, at Memingen; Physician at Hamburgh


### *Case of Amaurosis of both Eyes, of Twelve months standing, successfully cured by* Mr. H. John Rheineck, Surgeon, at Memingen.

(Communicated by Dr. Von Embden, Physician at Hamburgh, in a Letter to Dr. Shearman.)

THE following interesting morbid history, affords a convincing proof of what may be effected by medicine, provided the morbid cause be clearly discovered, and shows how material its investigation is in attempting a cure. Patients very often, particularly in cases like that before us, obstinately deny their former diseases, by which the practitioner is not only kept in the dark, but they themselves are deprived of all hopes of recovery.

That amaurosis very often belongs to those kinds of blindnes which are incurable, and where the practitioner not seldom exhausts in vain his fund of knowledge and remedies, experience has long shown. That there are instances, however scarce they may be deemed, where medical aid may succeed, the following case will evince.

In the year 1798, the author, then being surgeon to the company of noble cadets at Berlin, one day accidentally went to his tailor, Mr. Wartenberg, who mentioned that his journeyman, Lidicke, had just been discharged as incurable, from the Dispensary for Diseases of the Eyes. Seeing the poor stone blind man sitting in the dining room bemoaning his fate, he went to him and examined him. He was a young man of about 24 years. Finding both his eyes apparently free from defect, the pupils moveable, yet deprived of all impression of light for twelve months past; he heard that his former tutor, the surgeon general Mr. Mursina, then the first practical oculist at Berlin, had used the best remedies against this disorder; of which belladonna, pulsatilla, arnica, valeriana, aconitum, camphor, electricity, g. guaiacum, g. ammoniacum, sal vol. c. c. assafœtida, tartar. emetic ranked amongst the first, but all to no purpose. The blind man assured him farther, that a year ago, he had lost his sight in the course of eight days. Not being able to find out any particular cause of the disease, notwithstanding the most exact examination, he requested the patient to let him examine his whole body undressed. This being done, several vestiges and scars of recently healed chancres were found about the genitals; he now inquired whether the patient had had any buboes, and was informed, that shortly before his becoming blind, he had some ulcers in the places just described; and also great swellings in the groins, which, however, had been quickly cured by the surgeon of a company. The author now rejoiced at having found out the cause, and assured the blind man, he might hope to recover his sight. He directly ordered him some medicine, consisting of mercurius solubilis Hanemani gr. x. opium gr. x. and camphor gr. xl; desiring him to shew the prescription to his former oculist Mr. Mursina, and to ask his opinion whether he should use it or not. The blind man being led the following day to the surgeon general, this worthy man rejoiced at the cause having been discovered, again received the patient into the hospital, ordered the medicine prescribed by the author to be continued, and in twenty days, the patient left the hospital, and went to work again; his sight being perfectly restored, after it had been lost a whole twelvemonth.

April 24th, 1816.

---

### THE REVIEWER TO DR. YEATS.

